# Decision Science Can Inform Clinical Trade-Offs Regarding Cardiotoxic Cancer Treatments

**DOI:** 10.1093/jncics/pkab053

**Published:** 2021-06-24

**Authors:** Arielle S Gillman, Jacqueline B Vo, Anju Nohria, Rebecca A Ferrer

**Affiliations:** 1 Division of Cancer Control and Population Sciences, Cancer Prevention Fellowship Program, Basic Biobehavioral and Psychological Sciences Branch, Behavioral Research Program, National Cancer Institute, Bethesda, MD, USA; 2 Division of Cancer Epidemiology and Genetics, Cancer Prevention Fellowship Program, Radiation Epidemiology Branch, National Cancer Institute, Bethesda, MD, USA; 3 Cardio-Oncology Program, Dana-Farber Cancer Institute and Brigham and Women’s Hospital, Boston, MA, USA; 4 Division of Cancer Control and Population Sciences, Basic Biobehavioral and Psychological Sciences Branch, Behavioral Research Program, National Cancer Institute, Bethesda, MD, USA

## Abstract

Cancer treatment-related cardiotoxicity (ie, heart failure, coronary artery disease, vascular diseases, arrhythmia) is a growing cancer survivorship concern within oncology practice; heart disease is the leading cause of noncancer death in cancer survivors and surpasses cancer as the leading cause of death for some cancers with higher survival rates. The issue of cardiotoxicity introduces a critical tradeoff that must be acknowledged and reconciled in clinical oncology practice: treating cancer aggressively and effectively in the present vs preventing future cardiotoxicity. Although many cancers must be treated as aggressively as possible, for others, multiple treatment options are available. Yet even when effective and less cardiotoxic treatments are available, they are not always chosen. Wariness to choose equally effective but less cardiotoxic treatment options may result in part from providers’ and patients’ reliance on “cognitive heuristics,” or mental shortcuts that people (including, research shows, medical professionals) use to simplify complex judgments. These heuristics include delay discounting, availability and affect heuristics, and default bias. In the current commentary, we describe relevant research that illuminates how use of heuristics leads to biased medical decision making and translate how this research may apply when the tradeoff between aggressive cancer treatment and preventing future cardiotoxicity is considered. We discuss the implications of these biases in oncology practice, offer potential solutions to reduce bias, and call for future research in this area.

Cancer treatment-related cardiotoxicity (or cardiovascular toxicity, ie, heart failure, coronary artery disease, vascular diseases, arrhythmia) is a growing cancer survivorship concern within oncology practice. Though advances in cancer treatment have contributed to improved cancer-specific survival rates over the past several decades (1-3), these improvements often involve a cost: adverse effects of cancer treatment, including cardiovascular side effects or cardiotoxicity from some treatments. Cardiotoxicity can affect patients acutely (during treatment) or late (years after completion of treatment), causing substantial declines in quality of life and increases in cardiovascular morbidity and mortality. Treatments with well documented risk of cardiotoxicity include anthracyclines (eg, doxorubicin, epirubicin), radiation to the chest wall, and trastuzumab (4). Using these cardiotoxic treatments in combination can increase risk considerably. Notably, heart disease is the leading cause of noncancer death in cancer survivors and has begun to approach or surpass cancer as the leading cause of death for some cancers with higher survival rates (5).

Accumulating evidence shows that anthracycline-induced cardiotoxicity affects nearly 6% to 18% (6) of patients and can be seen up to 40 years after treatment completion (7). Heart disease risk increases with greater cumulative lifetime dose of anthracyclines (8). Further, the risk of heart disease increases when anthracyclines are used concomitantly with trastuzumab or radiation (4). Heart disease resulting from radiation therapy is often seen 5 years after treatment completion and increases linearly with mean radiation doses to the heart (9). Although cardiotoxicity may occur acutely, it is important to note the impact of late effects that arise long after treatment has ended.

Risk of cardiotoxicity introduces a critical tradeoff that must be acknowledged and reconciled in oncology practice: treating cancer aggressively and effectively in the present vs preventing cardiotoxicity in the future. Although many cancers must be treated as aggressively as possible, often including the use of potentially cardiotoxic treatments, for others, multiple treatment options can be effective.

For example, early-stage, HER2-negative breast cancer patients may receive either: 1) doxorubicin or epirubicin and cyclophosphamide [AC], or 2) docetaxel and cyclophosphamide [TC]. Recent trials have demonstrated only marginal improvement (10) or no difference (11) in breast cancer survival with AC vs TC, which is important because AC carries substantial increased risk of cardiotoxicity compared with TC. As another example, for early-stage, HER2-positive, lymph node–positive breast cancers, adjuvant treatment may consist of: 1) doxorubicin, cyclophosphamide, paclitaxel, trastuzumab, and pertuzumab [AC-THP]; or 2) docetaxel, carboplatin, trastuzumab, and pertuzumab [TCHP]. Recent studies demonstrated no difference in breast cancer survival rates by inclusion of anthracyclines (12–14), but AC-THP has a substantially higher risk of cardiotoxicity due to the combination of anthracyclines with trastuzumab (15,16). Because both regimens contain trastuzumab, a targeted therapy aimed at inhibiting the HER2 receptor, a less cardiotoxic option would be selecting TCHP and thereby removing the additional risk of cardiotoxicity due to anthracyclines. Of course, breast cancer is a heterogenous disease, and avoiding cardiotoxic treatments in certain cases is not possible (eg, triple-negative breast cancer) (17). However, when the opportunity is available, selecting a less or noncardiotoxic option would minimize cardiotoxicity risk later.

Additionally, treatment of favorable-prognosis, early-stage Hodgkin lymphoma typically involves either: 1) doxorubicin, bleomycin, vinblastine, dacarbazine [ABVD] chemotherapy followed by chest radiation; or 2) ABVD alone. Although radiation after ABVD has demonstrated benefits for disease-specific survival, these benefits may be offset by delayed cardiotoxic effects (18) (up to 20 years after radiation) (19), the risk for which is greater when radiation is combined with anthracycline chemotherapy (19–21). Here, omitting chest radiation is an option to minimize the risk of delayed cardiotoxicity.

Factors at multiple levels of the health-care system can affect cancer treatment choices, including treatment availability, cost, insurance coverage, and clinical guidelines. Yet, critically for the purposes of this commentary, there is also a “human factor” in the decision-making process; typically, the oncologist presents the patient with multiple treatment options, risks and benefits of each are discussed, and a decision is reached over which treatment to ultimately proceed with (22–24). Within this decision-making process, oncologists and patients alike may be wary of forgoing use of aggressive yet cardiotoxic cancer treatments, even when other evidence-based treatments are available, as evidenced by the continued use of cardiotoxic therapies in cases such as the examples described above. We propose that this wariness may result in part from reliance on “cognitive heuristics,” or mental shortcuts that people use to simplify complex judgments (25). Certain heuristics are known to affect provider and patient medical decision making (26) and are particularly relevant to the features of this particular tradeoff. These include: 1) delay discounting, 2) availability and affect heuristics, and 3) default bias. Reliance on heuristics is often adaptive in that they allow for quick, efficient decision making and often lead to advantageous outcomes. However, there is a wide literature base demonstrating that reliance on heuristics can, in some circumstances, lead to biased decision making, including among physicians (27–30). Moreover, in some contexts, experts may be even more susceptible to decision-making biases than novice decision makers (31). Translating established, empirical research on these biases to help understand tradeoff decisions involving cardiotoxic cancer treatments can help guide more informed decisions in this context. Importantly, even decisions influenced by complex, multilevel factors can be improved when efforts are made to reduce reliance on cognitive heuristics (32–34). Below, we review the decision science literature to identify principles that should be taken into account when informing optimal decision making in this context.

## Delay Discounting Bias

Individuals tend to favor immediate over delayed outcomes (35) when given such an “intertemporal choice”—a bias termed “delay discounting.” Deciding whether to prescribe or use cancer treatments known to have late-onset cardiotoxic effects is a classic example of intertemporal choice (ie, choosing the immediate outcome of aggressively treating cancer now to reduce a health risk in the present vs the delayed outcome of preventing cardiotoxicity to reduce a health risk in the future). Conceptualizing the reduction of a health risk as an outcome that can be traded off over time may be somewhat counterintuitive compared with something tangible, such as money, because individuals cannot guarantee good health in either the present or future—they can only influence the probability of being in good health. That said, individuals still demonstrate present-time biases for health tradeoffs as they do with tangible monetary rewards (36).

The tendency to overvalue present outcomes is exacerbated when the time delay between present and future outcomes is larger and when present outcomes are seen as equivalent or greater than future outcomes but can occur even when future gains severely outweigh present gains (35). Moreover, some individuals are more susceptible to this bias than others. These individual differences in preferences for immediate vs delayed outcomes may predict cancer treatment preferences of patients (37) and clinical decisions by providers (38,39). Additionally, people have difficulty imagining how they will feel in the future in response to an affectively laden event they are not experiencing in the present (deemed the “hot-cold empathy gap”) (40). This may exacerbate delay discounting in this context, because patients and providers are unlikely to be able to fully imagine how they would feel in the future if today’s cancer treatment were to create cardiotoxic late effects.

Although it is an understandable human desire to fix an immediate problem now and delay consideration of the consequences of that decision until necessary, given the clear evidence regarding the prevalence and seriousness of cardiotoxic late effects of certain cancer treatments, oncology providers should be aware of delay discounting bias when making treatment decisions.

## Availability and Affect Heuristics

Despite the fact that heart disease is more common than cancer, approximately one-half of Americans believe that cancer is more common than heart disease, and nearly 80% believe their own risk of cancer is higher than or equal to their risk of heart disease (41). Individuals also tend to report believing cancer is a more common cause of death than heart disease (42). This is important because risk perceptions are a key predictor in models of medical decision making (43), and if individuals have inaccurate perceptions about the risk of heart disease compared with cancer, they are unlikely to take steps to mitigate that risk. Several potential explanations may contribute to inaccurate risk perceptions for heart disease vs cancer. One is the “availability heuristic,” through which people judge outcomes as more likely if they are able to retrieve greater instances of that outcome from memory (44). Media coverage of celebrity cancer deaths and exposure to awareness campaigns may contribute to incorrect public perceptions that cancer risk is higher than heart disease risk (45). Also underlying these imbalanced risk perceptions is the “affect heuristic”; people use their emotions to inform their judgments of personal susceptibility, and cancer is considered a uniquely frightening disease (46,47).

Although one might expect that medical professionals would be less susceptible to biased risk perceptions, research suggests otherwise (48). Moreover, medical oncologists who are exposed daily to the reality of a cancer diagnosis think of heart disease risk comparatively less often, which could exacerbate availability biases related to cancer as well as reduce affective risk perceptions for heart disease. These biased comparative risk perceptions could cause imbalances in the way providers weight the potential of cardiotoxic late effects with their perception of the need to eradicate cancer as aggressively as possible. The fact that oncology providers consider delayed consequences pertaining to cancer (eg, prescribing adjuvant hormonal therapy to women with early-stage breast cancer to reduce the risk of cancer recurrence) but often do not extend the same logic to cardiotoxicity suggests that there is indeed some degree of imbalance in the way that cancer vs heart disease risks influence decision making.

Similarly, biased comparative risk perceptions among patients may render them less receptive to less aggressive (but perhaps similarly effective and less cardiotoxic) therapies and bias how they communicate such preferences with their providers. In line with this assertion, a recent study found that healthy participants were more willing to use a hypothetical medication that reduced cancer risk and increased heart disease risk than if that medication reduced heart disease risk and increased cancer risk by the same amount (49). Although this decision was hypothetical, it nonetheless sheds light on the very real potential that biased risk perceptions could be consequential for patient decisions.

Availability bias can affect medical decision making in consequential ways outside of risk perceptions. For example, when asked to provide primary treatment recommendations for a prostate cancer case scenario, radiation oncologists and urologists were more likely to recommend primary treatments that favored their specialty and to report believing treatment in line with their specialty would lead to better quality of life for the patient (29). These findings are likely due in part to availability bias, and it is reasonable to think that specialty bias could also apply to clinical oncologists without overlapping expertise in cardiology. In fact, a recent survey of cardiologists and oncologists found that cardiologists were much more likely than oncologists (55.8% vs 12.5%) to believe it was important to monitor cancer patients for cardiotoxicity even in the absence of signs or symptoms, suggesting that specialty bias likely does play a role in clinical decision making in this context (50).

Thus, biases resulting from overreliance on availability or affect heuristics may implicitly or explicitly affect tradeoffs guiding treatment decisions in oncology.

## Default Bias

When individuals make choices that involve a default option, they are much more likely to choose to maintain the status quo (51), including among physicians and for consequential medical decisions (termed default bias or status quo bias) (52). Individuals likely gravitate toward default options because defaults are viewed as normatively preferred and because deviating from the default involves effort and increased personal responsibility (53). Cardiotoxic treatments can be conceptualized as the default treatment option for several cancers; anthracyclines have been a critical element of many chemotherapy regimens since the 1980s (54) and radiation therapy for longer.

Thus, wariness towards less cardiotoxic treatment alternatives may reflect providers’ resistance to change (54) and preferences to support existing default or status quo options. This desire is also likely to be transferred to the patient; patients are much more likely to choose the option presented to them by their provider as the default (55). Moreover, default bias becomes stronger in medical decisions when more alternatives are added to the set of choices (52). As such, default bias may be increasingly relevant as new alternatives to cardiotoxic regimens become available (27). Thus, when effective alternatives to treatments with risk of cardiotoxicity exist, it may be prudent for those options to be widely endorsed by the oncology community as the default (a shift in default treatment options may already be occurring in some cases, evidenced by declining use of anthracyclines over time for certain cancers) (56).

## Practical Implications

### Clinical Guidelines for Preventing Cardiotoxicity: Increasing Awareness and Adherence

The oncology community has begun to reckon with the tradeoffs involved in treatment decision making when cardiotoxicity risk is involved (57), yet there are limited data to support decision making at this point of care. Considering how best to make these tradeoffs is of growing importance in clinical oncology practice. For example, increasing awareness of treatment-related cardiotoxicity has contributed to the development of clinical guidelines, which can serve 2 purposes: 1) increasing awareness of cardiotoxicity among oncologists and patients to facilitate treatment decision making regarding potential cardiotoxic risk; or 2) in the case where the more cardiotoxic treatment option is necessary, facilitating the care and management of patients who receive cardiotoxic treatment.

In 2017, the American Society of Clinical Oncology (ASCO) released clinical practice guidelines as one of the first organizations to address how to care and manage cardiovascular disease specifically related to cancer treatment. Recommendations include identifying patients at high risk by treatment type: 1) high-dose anthracyclines, 2) high-dose radiation, 3) combination of lower-dose anthracycline with lower-dose radiation, 4) trastuzumab with anthracycline, and 5) anthracycline or trastuzumab with preexisting cardiovascular risk factors (58). Critically, ASCO recommends avoiding or minimizing use of cardiotoxic treatments if alternatives exist that do not affect cancer-specific outcomes. Another recommended strategy to minimize risk of cardiotoxicity includes limiting lifetime cumulative dose of anthracyclines (doxorubicin 400 mg/m^2^, epirubicin 600 mg/m^2^) due to increasing cardiotoxicity risk with higher doses.

In situations where cardiotoxic cancer treatment outweighs alternatives and must be used, it is important to manage the patient’s cardiovascular disease risk. At diagnosis, it is crucial to assess and manage preexisting cardiovascular risk factors, including smoking, hypertension, dyslipidemia, obesity, and diabetes. When administering chest radiation, clinicians should use the lowest effective radiation dose and use precise therapy to limit exposure to the heart (eg, deep breath hold to shift the heart out of the radiation field and intensity-modulated radiation). Before, during, and after treatment, oncologists should collaborate with cardiologists or cardio-oncologists to ensure appropriate monitoring of cardiac function in high-risk patients. Importantly, some of the biases we discuss above in relation to the treatment decision-making process (eg, inaccurate risk perceptions for heart disease) may also partially explain poor adherence to guidelines for monitoring cardiac function in patients treated with cardiotoxic treatments (59,60).

Lastly, 2 other frameworks are available to increase understanding and knowledge of cardiotoxicity risk. The National Comprehensive Cancer Network has a framework for assessing risk of cardiotoxicity in patients who have received anthracycline therapy. Incorporating the framework into electronic health records to prepopulate risk or management strategies could be an option to minimize long-term effects (57). Another strategy is the development of the ABCDE steps to prevent heart disease in breast cancer patients (61). This tool could increase awareness for both oncology providers and patients to understand risk factors and cardiovascular symptoms through an acronym: awareness of risks for heart disease, aspirin, blood pressure, cholesterol, cigarette and tobacco cessation, diet and weight management, dose of chemotherapy, diabetes management, exercise, and echocardiogram. Providers also should assess patients’ understanding and perception of cardiovascular disease severity and potential side effects (ie, shortness of breath, fatigue, swelling) (4), keeping in mind that patients’ existing perceptions are likely affected by the biased processes we discuss in this commentary, including inaccurate risk perceptions for heart disease vs cancer that stem from affect and availability heuristics.

Despite recent development of guidelines and evidence that early detection and intervention can mitigate cardiac damage from cancer treatment, in a recent study, only 65% of oncologists reported that they refer to clinical guidelines for decision making and only 12% of oncologists believe that cardiotoxicity should be monitored in asymptomatic patients (50). Therefore, ensuring awareness of clinical guidelines and using multidisciplinary collaboration is important to provide holistic care of the cancer patient. Moreover, the growing field of cardio-oncology, which is made up of experts and providers who are well versed in the intersection of oncology and cardiology, is well positioned to address these issues. Such providers are less likely to demonstrate biases in favor of treating cancer at the cost of cardiac health and may be able to provide more objective guidance for high-risk patients. Unfortunately, cardio-oncology clinics are currently concentrated in large academic centers but are not prevalent in community cancer centers, which is likely to exacerbate disparities until these clinics can reach a wider group of patient populations.

Long-term survival of cancer survivors highlights the need for care beyond oncology. Primary care providers are uniquely positioned to provide long-term continuity of care (62). Primary care providers can monitor cardiovascular risk factors, obtain cardiovascular monitoring as indicated based on treatment, and facilitate patients’ uptake of healthy lifestyle recommendations to reduce modifiable cardiovascular risk factors (63). Yet primary care providers must be made aware of and acknowledge the potential cardiotoxic effects of cancer treatment. In 2016, Runowicz and colleagues (62) published breast cancer survivorship guidelines on behalf of the American Cancer Society and ASCO to provide recommendations for primary care clinicians. Successful multidisciplinary collaboration between oncologists, cardiologists and cardio-oncologists, and primary care providers can help mitigate late- and long-term cardiovascular effects of cancer treatment.

### Opportunities for Decision Science to Inform Clinical Practice

As we have highlighted in this commentary, decision-making biases are consequential in medical decision making and may implicitly or explicitly contribute to providers’ and patients’ resistance to limiting use of certain cancer treatments despite evidence of their cardiotoxic effects. Although there are situations in which such treatments are necessary, when effective noncardiotoxic alternative treatments are available, yet not chosen, we suggest that clinical oncology practitioners consider how these decision-making biases play a role in such decisions and attempt to counter any unnecessary bias. Importantly, people may be more likely to use heuristics with outgroup (eg, those outside one’s racial, ethnic, sex, or nationality group) compared with ingroup members (64), so these biases may exacerbate health inequities in cardiovascular outcomes among cancer survivors (65).

Given how these biases might lead to challenges in cardiotoxicity-related decision making, what can be done to guide patients and providers toward better choices? An early step is educating the medical oncology community about biases, which we have attempted to initiate in this commentary and which has been shown to improve doctors’ decision making in certain contexts (27). Importantly, research suggests that physician motivations are likely to (implicitly or explicitly) drive shared decision making for cancer treatments more so than patient motivations and preferences (66–68). Further, patients often rely on providers to qualitatively interpret numerical risk estimates for them; that is, if the risk of a side effect for a medication is 10%, patients will typically turn to their provider’s interpretation when deciding whether a numerically represented risk estimate is “high” or “low” (69). Thus, it is the role of the provider to transparently discuss risks and benefits of treatment options when relevant, help patients make meaningful interpretations of risk estimates, ensure their comprehension, and allow patients to take time to absorb complex information before coming to a decision. Taking care to clearly explain (and not downplay) risks of cardiotoxicity to patients may be critical.

However, education about biases only improves decision making to a degree (32). Unfortunately, there is currently a lack of high-quality interventions that attempt to formally reduce cognitive biases among providers (70). Nevertheless, decision science research suggests some additional potential solutions, including the use of cognitive strategies to reduce bias (32). Interventions that help individuals better predict how they will react to a future decision can reduce the hot-cold empathy gap that is involved in delay discounting errors (71). Delay discounting may be mitigated in other ways, for example, through helping individuals orient their attention to the future (72). Findings such as this might be translated to clinical practice through prompting patients to consider how the treatment decisions they make will affect them both in the short term (eg, 1-5 years) and long term (eg, 10 or 20 years from now), and similarly, through prompting clinicians to focus more attention onto long-term consequences of treatment decisions for their patients in addition to shorter-term consequences (73). Mindfulness-based interventions, which have wide-ranging benefits for cancer survivors outside of the treatment decision making context (74,75), may also help to reduce delay discounting bias, as reported in a systematic review (73).

With regard to reduction of bias caused by the availability and affect heuristics, 1 solution is through the use of evidence-based methods of risk communication to characterize the risk of cancer vs cardiotoxicity risks of different treatment options. For example, decision aids are commonly used in the field to support shared decision making in cancer care. However, we caution that the majority of studied decision aids have been developed without specific grounding theories in decision science, which may explain their mixed effects on health-related outcomes (76). Moving forward, knowledge about biases should be incorporated when developing shared treatment decision-making tools, and empirically supported strategies to combat these biases should be used. For example, risks surrounding cancer treatments can be better understood if they are communicated using evidence-based methods, such as through the use of pictographs (77).

Finally, the power of defaults can be harnessed positively by the medical oncology community by ensuring that the most cardiotoxic treatments are not perceived as the default option if there is a reasonable, less cardiotoxic alternative and instead shifting the default toward treatment options that are less cardiotoxic or noncardiotoxic (53). Such a shift is less about individual-level communication on part of the provider and more of a shift at the systemic level, which is also a critical approach to the problem. Implementing the clinical practice guidelines surrounding cardiotoxicity that we discussed above is 1 way to begin a broad shift in oncology toward the prevention of cardiotoxicity.

## Summary

We have outlined how several well-researched judgment and decision-making biases may affect clinical decisions related to cancer treatments with cardiotoxic effects based on research in other domains, medical or otherwise, which we expect should generalize to this context. However, no current empirical research that we know of has examined the extent to which these or other biases may affect decision making in this specific context, nor whether interventions to reduce cognitive biases may improve outcomes in this domain. This topic is a critical avenue for future research. Additionally, these processes likely generalize to decisions about other cancer treatments that involve temporal tradeoffs. For example, treatment decision making involves weighing immediate effectiveness of treatments with other long-term risks and toxicities, including second cancers or cognitive impairments (78). Similarly, cancer treatments that may have reproductive effects involve a similar time-related tradeoff, where individuals may have to weigh the benefits of beginning cancer treatment immediately vs delaying cancer treatment slightly to take measures to preserve long-term fertility in adolescent and young adult cancer survivors (79,80). In sum, decision science can illuminate how clinical tradeoffs in oncology are navigated and ultimately help survivors live healthy and long lives after cancer.

## Funding

Not applicable.

## Notes

**Role of the funder:** Not applicable.

**Disclosures:** Anju Nohria discloses financial relationships: Amgen, Inc (research support) and Takeda Oncology (DSMB—consultant). Arielle Gillman, Jacqueline Vo, and Rebecca Ferrer have no disclosures to report.

**Author contributions:** Conceptualization: ASG, JBV, RAF; Writing—original draft: ASG, JBV; Writing—review and editing: ASG, JBV, RAF, AN; Supervision: RAF, AN.

**Disclaimer:** The opinions expressed by the authors are their own and this material should not be interpreted as representing the official viewpoint of the U.S. Department of Health and Human Services, the National Institutes of Health or the National Cancer Institute.

**Acknowledgements:** Anju Nohria is supported by the Catherine Geoff Fitch fund and the Gelb Master Clinician fund.

## Data Availability

Not applicable.
